# Clinical complete responders to definite chemoradiation or radiation therapy for oesophageal cancer: predictors of outcome

**DOI:** 10.1186/1471-2407-13-413

**Published:** 2013-09-06

**Authors:** Antoine Adenis, Emmanuelle Tresch, Sylvain Dewas, Olivier Romano, Mathieu Messager, Eric Amela, Stéphanie Clisant, Andrew Kramar, Christophe Mariette, Xavier Mirabel

**Affiliations:** 1 Gastrointestinal Oncology Department, Centre Oscar Lambret, 3 rue Combemale, 59020 Lille Cedex, France; 2 Catholic University, 60 Boulevard Vauban, 59800 Lille, France; 3 Methodology and Biostatistics Unit, Centre Oscar Lambret, 3 rue Combemale, 59020 Lille Cedex, France; 4 Department of Radiotherapy, Centre Oscar Lambret, 3 rue Combemale, 59020 Lille Cedex, France; 5 Lille University, Faculté de Médecine Henri-Warembourg, Lille Cedex 59045, France; 6 Surgery Department, University Hospital, Hôpital C Huriez, Place de Verdun, 59037 Lille Cedex, France; 7 Clinical Research Unit, Centre Oscar Lambret, 3 rue Combemale, 59020 Lille Cedex, France

**Keywords:** Oesophageal cancer, Chemoradiotherapy, Radiation therapy, Prognosis

## Abstract

**Background:**

To identify predictors of long-term outcome for patients with clinical complete response (cCR) after definite chemoradiotherapy (CRT) or radiation therapy (RT) for oesophageal cancer (EC).

**Methods:**

In this retrospective study, we reviewed the files of all patients from our institution that underwent definitive RCT or RT for EC, from January 1998 to December 2003. Among 402 consecutive patients with EC, 110 cCR responses were observed, i.e. without evidence of tumour on morphological examination of the biopsy specimens, 8 to 10 weeks after radiation. Baseline patient and tumour characteristics were as follows: male = 98/110, median age = 60, squamous histology = 103/110, tumour site (upper/middle/lower third) = 41/50/19, weight loss none/<10%/≥10% = 36/45/29, dysphagia grade 1/2/≥3 = 30/14/66. Patients were staged according to endosonography and/or computed tomography. There were 9 stage I, 31 stage IIA, 15 stage IIB, 41 stage III, 6 stage IV. Post treatment nutritional characteristics were as follows: weight loss during treatment none/<10% ≥ 10% = 35/38/37, remaining dysphagia grade 1/2/≥3 = 54/24/32. Univariate and multivariate analyses were performed using log-rank and Cox proportional hazards models, and survival curves were estimated using the Kaplan-Meier method.

**Results:**

During follow up (median: 6 [0.4–9.8] years), 16 patients had salvage surgery. Median OS was 2.5 years, and 5-year OS was 33.5%. Histological type, stage, age, gender, and treatment characteristics had no significant impact on outcome. The risk of death was increased two-fold for patients with grade ≥ 3 dysphagia after treament (HR = 1.9 [1.2–3.1], p = 0.007). Weight loss ≥10% during treatment also negatively affected outcome (HR = 1.8 [1.0–3.2], p = 0.040).

**Conclusion:**

One EC patient among 3 with cCR after definite CRT/RT is still alive at 5 years. Variables related to reduced OS were: remaining significant dysphagia after treatment and weight loss ≥10% during treatment.

## Background

Oesophageal cancer (EC) is a devastating malignancy which ranks 6^th^ on the list of cancer-mortality causes [1]. Pooled data from European registries indicate that 1-year and 5-year overall survival (OS) rates are 33% and 10%, respectively [2].

Surgery continues to be the mainstay of treatment for patients with localised and locally advanced EC in the absence of medical contraindications [3] with a 5-year survival rate as high as 47% in a large-volume referral centre [4]. With respect to survival outcome and also from an organ preservation standpoint some patients may benefit from a program excluding surgery since the pioneer publication of the Radiation Therapy Oncology Group (RTOG) [5] which showed that 26% (95% confidence interval (CI): 15%–37%) of patients treated with CRT (as compared to radiation only) were still alive at 5 years [6].

Because complete response (CR) [7,8] is a major predictor of outcome for EC treated with definite CRT, many efforts have been made these last years, to define just what CR corresponds to [9], and to increase the rate of CR with new CRT regimens [10,11]. CR assessed by endoscopy has been chosen by investigators in recent prospective trials [9,11] because of its validity as a good surrogate marker for OS [12,13], and because of the unsatisfactory accuracy of CT scan and endoscopic ultrasonography (EUS) in the restaging after CRT [13,14]. Moreover, the decision-making impact of endoscopic biopsies remains questionable, due to the high false negative rate of this procedure [15-17].

The CR rate as the primary endpoint of a prospective trial for evaluating treatment of patients with EC has not been specifically studied, and there is sparse data about the specific outcome of these patients other than the fact that they have a greater chance to survive. In surgical series, the rate of ypT0N0M0 (i.e. pathologic CR) after preoperative CRT is about 20–30%% [4,18,19], and this group of patients may achieve an excellent 5-year survival as high as 55%, with the best outcome for younger patients [19]. Using clinical tools–with their poor sensitivity–for clinical restaging, results from some studies showed that clinical CR (cCR) rates varied from 30% to 62% [10,11,20-22], depending on the time period of treatment, imaging modalities (with or without post treatment biopsies), and, obviously, on treatments. OS of patients with cCR has not been specifically addressed, and their determinants of outcome are mostly unknown.

In the current study, we reviewed our series of 110 patients with cCR who were treated with definite RCT or radiotherapy (RT) for EC, and staged by available clinical means, in order to identify prognostic factors that predict their long-term outcome.

## Methods

### Patient population

From January 1998 to December 2003, 402 consecutive patients with localized or locally advanced EC underwent definite CRT or RT at the Centre Oscar Lambret (Northern France Cancer Centre, Lille). One hundred ten patients with cCR (27%) formed the basis of this study, which was conducted according to the Helsinki declaration and the national French laws for retrospective monocentric studies (Commission Nationale Informatique et Liberté agreement 1034071, Sept 27^th^, 2004), and registered in http://clinicaltrials.gov (NCT01525953).

### Pretreatment evaluation

Pretreatment evaluation included physical examination, barium swallow, and endoscopy of the oesophagus, and thoracic and abdominal CT and/or EUS. Patients were classified with EUS according to the 1997 AJCC staging system [23], and/or with CT according with the so-called modified Wurtz classification [24] which makes a CT-defined T3 (ct-T3) a tumour whose largest diameter is over 30 mm, without suspicion of adjacent organ involvement. In this CT classification, lymph nodes are considered malignant if their largest diameter is over 10 mm.

Fluorodeoxyglucose positon emission tomography was not used. Dysphagia was evaluated according to the Atkinson’s classification: grade 1, ability to eat a normal diet; grade 2, ability to eat some solid food; grade 3, ability to eat some semisolids only; grade 4, ability to swallow liquids only; grade 5, complete dysphagia [25].

### Treatment details

RT was delivered with megavoltage equipment (> 8 MV) using a multiple field technique. Patients were treated 5 days per week and most of them received 1.8 Gy/d in 28 fractions (total dose: 50.4 Gy). The total RT dose to the spinal cord was limited to 40 Gy. Patients were treated through a 3 or 4 fields technique with all fields treated each day. Prescription doses were specified at the International Commission on Radiation Units and Measurements Report 50 reference point. The superior and inferior borders of the radiation fields were 3 cm beyond the primary tumor. The lateral, anterior, and posterior borders of the fields were 2 cm beyond the borders of the primary tumor. The primary and the regional lymph nodes were included into the radiation fields as were supraclavicular lymph nodes and celiac lymph nodes for tumors of the upper esophagus and lower esophagus, respectively.

All patients underwent the cytotoxic schedule of the so-called “RTOG regimen” [5] except those who were treated on a phase I protocol with weekly vinorelbine in conjunction with RT (64 Gy, 2 Gy per fraction) [26], and patients whose medical condition did not allow for 5-fluorouracil and/or platinum salts administration.

### Follow-up evaluation

Patients were planned to be re-staged 8 to 10 weeks after the end of radiation, that is more or less 3–4 weeks after the last chemotherapy dosing. Post treatment evaluation included physical exam, upper endoscopy plus biopsies and thoracic and abdominal CT scan. Complete clinical responders were defined as patients without evidence of tumour on physical examination, on endoscopy, on oesophageal biopsies, and on CT scan. Follow-up consisted of an upper endoscopy yearly, or earlier if clinically indicated. Subsequent thoracic and abdominal CTs were not routinely obtained unless clinically indicated. Patterns of treatment failure were defined as the first site of failure. Regional failure included the primary tumour and the regional lymph nodes. Follow-up data were obtained from medical records and referring physicians.

### Statistics

Patient characteristics were described with median and extreme values for continuous variables and with frequencies and percentages for categorical variables. All event times were calculated from the last day of radiation therapy. Survival was assessed with the Kaplan-Meier method. The influence of categorical variables on survival was investigated with the Log-Rank test for univariate analyses, and the Cox proportional hazards model was used for multivariate analyses. To obtain a prognostic score, an integer weight was assigned proportional to the regression coefficients of each significant variable obtained from the multivariate Cox model and then combined to obtain an overall score. The number of prognostic categories was grouped together using a hierarchal coding system. The predictive discrimination of the model was evaluated with Harrell’s C statistic.

## Results

### Patient and treatment characteristics

There were 98 men and 12 women, and age ranged from 37 to 85 years (median = 60). Patient and treatment characteristics are presented in Table 1. Most patients (74/110) presented with significant weight loss (WL) at baseline: 29 patients (26.4%) had lost more than 10% of their body-weight, 28 (25.5%), had lost between 6 to 10%, and 17 (15.5%) had lost less than 5%. 30/110 patients (27.5%) presented without any dysphagia (grade 1), and 14 (12.8%), 54 (49.5%), 8 (7.3%), and 3 (2.8%) presented with grade 2, grade 3, grade 4, or grade 5 dysphagia respectively. There were 9 stage I, 31 stage IIA, 15 stage IIB, 41 stage III, 6 nodal stage IV. In 8 cases, we were not able to retrieve enough good quality data to assess the tumour stage. Most of the patients received the RTOG regimen [5] with 50.4 Gy in 28 fractions, plus 2 cycles of concurrent chemotherapy (5-fluorouracil-cisplatin), then 2 cycles of sequential chemotherapy. Even though our patients were treated with the primary aim of definite CRT, there is a subset of 16 patients who subsequently had oesophagectomy for local recurrence (n = 3), or for some other reason (remaining dysphagia = 2; patient’s wish after open discussion with his surgeon = 13). Some patients (75/110) presented with significant WL at restaging (vs baseline): 37 patients had lost more than 10% of their body-weight, 25 had lost between 6 to 10%, and 13 had lost less than 5%. At restaging, 54/110 patients (49.1%) presented without any dysphagia, and 24 (21.8%), 18 (16.4%), 8 (7.3%), and 6 (5.5%) presented with grade 2, grade 3, grade 4, or grade 5 dysphagia respectively. An improvement in dysphagia was only seen in one half of the patients (55/110), while 22/110 patients (20.2%) presented with worsened dysphagia.

**Table 1:** Patients and treatment characteristics

**N = 110**	**n**	**%**
Gender		
M	98	89.1%
F	12	10.9%
Age		
≤ 60	60	54.5%
>60	50	45.5%
Histology		
Adenocarcinoma	7	6.4%
Squamous cell	103	93.6%
Tumour site		
Upper third	41	37.3%
Middle third	50	45.5%
Lower third	19	17.3%
Staging (CT or EUS) (n = 102)		
I	9	8.8%
IIA	31	30.4%
IIB	15	14.7%
III	41	40.2%
IV	6	5.9%
Radiation (Gy)		
<50.4	3	2.7%
50.4	58	52.7%
>50.4	49	44.5%
Chemotherapy		95	86.4%
Cisplatin + Fluorouracil	91	82.7%	
Other*	4	3.7%	
Surgery	16	14.5%	
For recurrence		3	2.7%
For remaining dysphagia		2	1.8%
Patient’s wish after open discussion with his surgeon, after chemoradiation		11	10%
Endoprosthesis	10	9.1%	
Before radiation		9	8.2%
After radiation		1	0.9%
Dilatation	16	14.5%	
Before radiation	14	12.7%	
After radiation	2	1.8%	

*carboplatin: 1, cisplatin: 1, vinorelbine: 2.

### Survival analysis, pattern of treatment failure, prognostic factors

**Figure 1:**
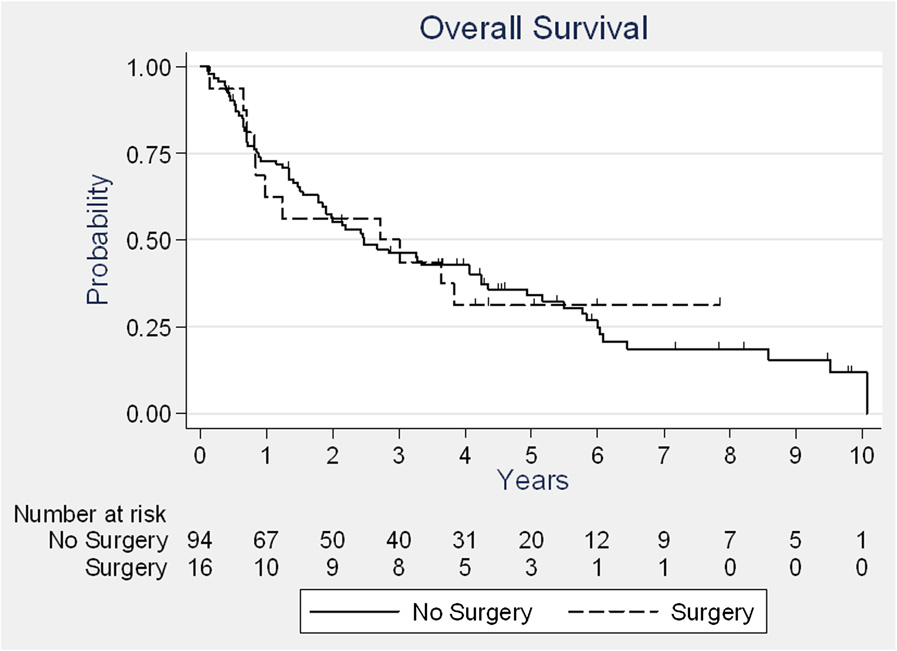
Overall survival according to surgery.

**Figure 2:**
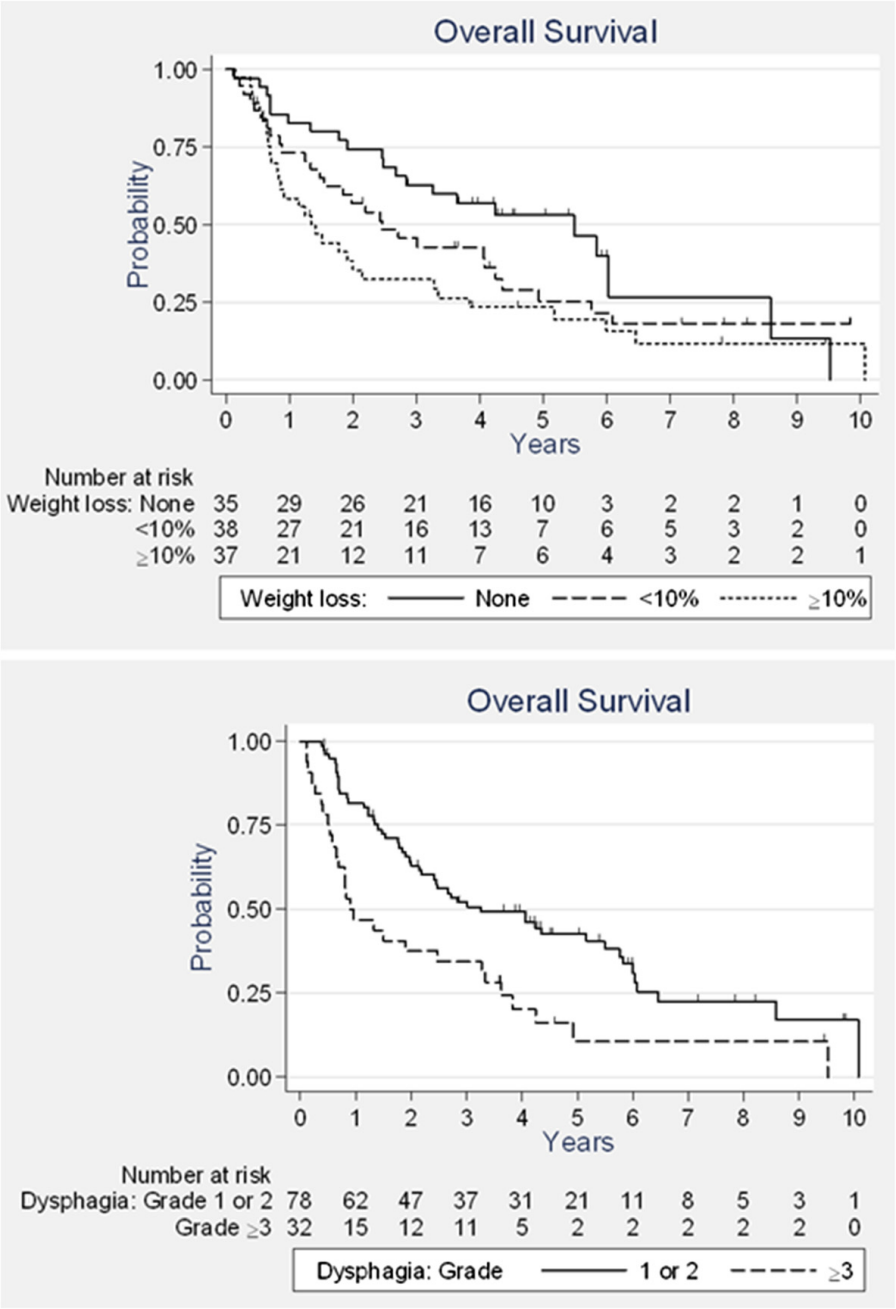
Overall survival according to weight loss during treatment (up), and grade of dysphagia after treatment (down).

With a median follow-up period of 6.0 years (range: 0.4 to 9.8 years), the median OS was 2.5 years. Three-and five-year OS rates were 46.9%, and 33.5%, respectively. A subgroup analysis of patients who had surgery (n = 16) revealed a median survival of 2.7 years (versus 2.5 years for the other 94 patients) (Figure 1). Forty-four patients (40%) experienced treatment failure. These recurrences were local only in 26 cases, distant only in 12 cases, and local and distant in 6 cases. Twenty recurrences (45.5%) occurred within the radiation field. Twenty-three patients (20.9%) experienced a second metachronous cancer. The presence of dysphagia after treatment, and WL during treatment were identified as significant predictors of poor OS in univariate and multivariate analysis (Table 2). The risk of death was increased two-fold for patients with weight-loss over 10% during treatment (HR = 1.8 [1.0–3.2], p = 0.04) (Figure 2) and for patients with grade ≥ 3 dysphagia after treatment (HR = 1.9 [1.2–3.1], p = 0.007). Histological type, stage, age, gender, weight-loss at baseline, and treatment characteristics did not show a significant influence on outcome. In our study, we were able to identify 3 groups of patients with different prognosis, depending on whether or not patients had, WL during treatment (score = 1 if WL < 10 and score = 2 if WL ≥ 10%) and/or remaining dysphagia after treatment (score = 2). Good prognosis patients had a score of zero (26 pts, median OS = 5.8), intermediate prognosis patients a score of 1 (28 pts, median OS = 2.7) and poor prognosis patients had a score of at least 2 (56 pts, median OS = 1.3). Harrell’s C index was equal to 0.656.

**Table 2:** Overall Survival according to prognostic factors (upper table: univariate analysis, lower table: multivariate analysis)

**Prognostic factors**		**N**	**n**	**Median OS [CI 95%]**	**p**	**Prognostic factors**		**N**	**n**	**Median OS [CI 95%]**	**p**
Total		110	80	2.5 [1.8–4.1]							
Age						Surgery					
	<= 60 y	60	39	2. 5 [1.8–6.0]	0.060		No	94	69	2.5 [1.8–4.2]	0.97
	>60 y	50	41	2. 7 [1.1–4.1]			Yes	16	11	2.7 [0.8–…]	
Gender						Albuminemia					
	M	98	71	2.5 [1.8–4.1]	0.92		<35 g/L	4	4	1.3 [0.5–…]	0.20
	F	12	9	2.0 [0.7–…]			≥35 g/L	46	34	3.3 [1.9–4.9]	
Histology						Dysphagia (baseline)					
	Adenocarcinoma	7	7	2.5 [0.6–4.1]	0.17		Grade 1–2	44	31	3.0 [1.8 – 4.2]	0.90
	Squamous cell	103	73	2.7 [1.8–4.2]			Grade ≥ 3	65	48	2.7 [1.5–4.4]	
Tumor site						Dysphagia (end of treatment)					
	Upper third	41	30	4.2 [2.0–5.8]	0.23		Grade 1–2	78	58	3.3 [2.1 – 5.5]	**0.0016**
	Middle third	50	35	1.9 [1.2–3.0]			Grade ≥ 3	32	28	0.9 [0.6–3.3]	
	Lower third	19	15	2.5 [0.7–3.3]							
Staging						Weight loss at baseline					
	I/IIA	40	28	3.3 [1.3–5.8]	0.074		None	36	22	5.2 [1.8–6.1]	0.20
	IIB/III	56	41	2.5 [1.5 – 3.8]			<10%	45	35	1.9 [1.2–3.0]	
	IV	6	6	0.9 [0.7–…]			≥10%	29	23	2.5 [1.3–3.6]	
Chemotherapy						Weight loss during treatment					
	No	15	11	2.0 [0.7–4.2]	0.36		None	35	21	5.5 [2.7–8.6]	**0.039**
	Yes	95	69	2.8 [1.8–4.1]			<10%	38	28	2.5 [1.5–4.2]	
Radiation dose							≥10%	37	31	1.4 [0.8–2.1]	
	≤50.4 Gy	61	41	3.3 [1.8–4.9]	0.45						
	>50.4 Gy	49	39	2.0 [1.1–4.2]							
**Prognostic variables**		**Deaths/N**		**OS rate [CI 95%] (3y)**		**Median OS [CI 95%] (y)**		**HR [CI 95%]**		**p**	**Score**
Weight loss during treatment											
None		21/35		63% [45–77]		5.5 [2.7–8.6]		1			0
<10%		28/38		46% [29–61]		2.5 [1.5–4.2]		1.5 [0.8–2.6]		0.18	1
≥10%		31/37		32% [18–48]		1.4 [0.8–2.1]		1.8 [1.0–3.2]		0.040	2
Dysphagia (end of treatment)											
Grade 1–2		52/78		52% [40–63]		3.3 [2.1–5.5]		1			0
Grade ≥3		28/32		34% [19–51]		0.9 [0.6–3.3]		1.9 [1.2–3.1]		0.007	2

## Discussion and conclusion

The main results from this long-term follow-up study with a prognostic factor analysis is that one EC patient out of 3 with cCR after definite CRT/RT is still alive 5 years after the end of treatment. This curative intent was achieved in a series of patients with mainly locally advanced EC. The bad news is that it occurred for a minority of patients only, in a subclass of EC who received definite CRT/RT. Our rate of cCR (27%) is a bit lower than what has been reported elsewhere (30 to 62%) [10,11,20-22], and may be reflecting differences in patient and tumour characteristics, as well as the intensiveness and timing of restaging work-up, and finally as to the true definition of cCR. Despite the recent and debatable input of fluorodeoxyglucose positron emission tomography [27,28] to standard work-up with morphological examination with biopsies, cCR remains difficult to assess accurately, because of the unsatisfactory sensitivity of CT and EUS, in the restaging after CRT [13,14]. Moreover, the negative predictive value of negative biopsies has been reported as comprised between 23% and 52% [15,29]. We were not able to reproduce the survival rates published by Ishihara et al. [20] in their series of 110 EC who achieved cCR after CRT (3-year OS: 66% vs our 47% [95% CI: 37.2–56%]), maybe due to patient selection or just because of a longer follow-up duration in our series. Obviously, we cannot compare our data to the excellent 55% 5-year OS rate obtained after preoperative CRT in ypT0N0M0R0 patients [19] selected on their ability to undergo surgery, and for whom the evaluation criteria (i.e. pathological CR) is far different from cCR.

It was disappointing for us to find that a majority of recurrences occurred within the radiation field, in this series of selected patients. We have to face the fact that it has been also reported elsewhere, either on retrospective or on prospective series [30-32].

This work is, to our knowledge, the first to report WL during treatment, and significant dysphagia after treatment as significant predictors of poor OS. It is implicitly known that remaining dysphagia after treatment may be related to persistent disease and implies a need for another treatment, even though it is difficult to distinguish it from an oesophageal stenosis caused by fibrosis or ischemic changes induced by RT. On that line, it is noteworthy that in the FFCD-9102 trial (CRT for locally advanced EC, then in case of clinical response, patients were randomised between CRT continuation or surgery) [33], some patients who were not randomised due to non response, no improved dysphagia, or other reason, were in pCR after subsequent surgery [34]. Therefore, remaining dysphagia after treatment does not seem a robust enough parameter for decision-making. DiFiore et al. [7] previously showed that baseline nutritional parameters (albumin serum level, body-mass index, dysphagia, or weight loss) were strong prognostic factors for survival, but in a series of patients treated with CRT, not in cCR only. We looked at the prognostic value of baseline albumin (data not shown), and we did not find any impact on survival. Because some of our patients had WL during treatment, we cannot rule out that our nutrition policy maybe was not watchful enough. On that line, some of our coauthors are strong advocates of artificial nutrition in patients undergoing CRT [35], although, the prognostic impact of nutrition has not yet been specifically addressed during CRT in that setting, When combining our 2 predictors of survival into a simple score, we obtained a doubling of median survival between the three groups for patients with none, one or two adverse factors. Whether or not this score may be use as a tool for decision-making, needs to be validated prospectively.

Obviously this study has some drawbacks. First, due to its retrospective nature, some clinical and biological data were not available in all cases, such as baseline CT and/or EUS staging, or baseline albumin or body-mass index. This missing information may have hampered the description of our cohort, and possibly the prognostic impact of some baseline characteristics. Second, the findings obtained from one center, in an area of rather high-incidence of squamous cells EC may not be directly extended to other countries, and needs to be refined in other settings. Third, we did not find any prognostic value of histology, stage, age or treatment characteristics, and we cannot rule out that it may be related to the small sample size of our series. Fourth, this is a long-term survival analysis, and some patients had been treated more that 13 years ago, at a period of time where our nutritional support policy was not as well defined as nowadays. Finally, our patients were not homogenously treated, although only a minority of patients received RT alone (15/110), or had a CRT regimen different from the one from the RTOG (4/95), or who had surgery (16/110) whereas it was not initially planned. Interestingly, 49/110 patients received higher radiation doses than the standard 50.4 Gy / 28 fractions schedule, but this factor had no prognostic impact in this series.

This work reinforces available evidence that some EC may be cured by RCT/RT only [6,31,33], even locally advanced ones. Whether or not patients may benefit from the correction of adverse prognostic factors such as weight loss during treatment, or dysphagia at the end of treatment by an intensive nutritional support during radiation or salvage surgery, respectively, needs to be prospectively assessed.

## Competing interests

The authors declare that they have no competing interests.

## Authors’ contributions

AA and XM conceived the study. SC gave administrative support. ET carried out the statistical analysis and wrote the statistical report under the supervision of AK. MM and CM provided patients and surgical data. AA, SD, EA, XM provided patients, and collected data. OR collected data and drafted the manuscript. AA wrote the manuscript with the support of XM and CM. All authors read and approved the final manuscript.

## Pre-publication history

The pre-publication history for this paper can be accessed here:

http://www.biomedcentral.com/1471-2407/13/413/prepub
